# Enhancing wound healing through innovative technologies: microneedle patches and iontophoresis

**DOI:** 10.3389/fbioe.2024.1468423

**Published:** 2024-10-28

**Authors:** Yong Xun Jin, Pham Ngoc Chien, Pham Thi Nga, Xin Rui Zhang, Nguyen Ngan Giang, Linh Thi Thuy Le, Thuy-Tien Thi Trinh, Shu Yi Zhou, Sun Young Nam, Chan Yeong Heo

**Affiliations:** ^1^ Department of Plastic and Reconstructive Surgery, College of Medicine, Seoul National University, Seoul, Republic of Korea; ^2^ Department of Plastic and Reconstructive Surgery, Seoul National University Bundang Hospital, Seongnam, Republic of Korea; ^3^ Korean Institute of Nonclinical Study, Seongnam, Republic of Korea; ^4^ Department of Medical Device Development, College of Medicine, Seoul National University, Seoul, Republic of Korea; ^5^ Department of Biomedical Science, College of Medicine, Seoul National University, Seoul, Republic of Korea

**Keywords:** CELLADEEP patch, wound healing, inflammation, microneedle therapy system, iontophoresis

## Abstract

**Introduction:**

Wound healing is a complex process involving multiple stages, including inflammation, proliferation, and remodeling. Effective wound management strategies are essential for accelerating healing and improving outcomes. The CELLADEEP patch, incorporating iontophoresis therapy and microneedle technology, was evaluated for its potential to enhance the wound healing process.

**Methods:**

This study utilized a full-thickness skin defect model in Sprague-Dawley rats, researchers compared wound healing outcomes between rats treated with the CELLADEEP Patch and those left untreated. Various histological staining techniques were employed to examine and assess the wound healing process, such as H&E, MT and immunofluorescence staining. Furthermore, the anti-inflammatory and proliferative capabilities were further investigated using biochemical assays.

**Results:**

Macroscopic and microscopic analyses revealed that the CELLADEEP patch significantly accelerated wound closure, reduced wound width, and increased epidermal thickness and collagen deposition compared to an untreated group. The CELLADEEP patch decreased nitric oxide and reactive oxygen species levels, as well as pro-inflammatory cytokines IL-6 and TNF-α, indicating effective modulation of the inflammatory response. Immunofluorescence staining showed reduced markers of macrophage activity (CD68, F4/80, MCP-1) in the patch group, suggesting a controlled inflammation process. Increased levels of vimentin, α-SMA, VEGF, collagen I, and TGF-β1 were observed, indicating enhanced fibroblast activity, angiogenesis, and extracellular matrix production.

**Discussion:**

The CELLADEEP patch demonstrated potential in promoting effective wound healing by accelerating wound closure, modulating the inflammatory response, and enhancing tissue proliferation and remodeling. The CELLADEEP patch offers a promising non-invasive treatment option for improving wound healing outcomes.

## 1 Introduction

According to current estimates, the prevalence of non-healing wounds is between 1% and 2% in wealthy countries, but it is much greater in underdeveloped nations. It is estimated that the yearly cost of wound care represents 3% of total healthcare expenses. Wound care has become a major global public health concern (Lindholm and Searle, 2016; Nussbaum et al., 2018; Martinengo et al., 2019; Holzer-Geissler et al., 2022). In addition to being expensive, wound treatment involves intricate processes involving different cell types, chemokines, and growth factors to mend skin injuries. Numerous health problems, especially protracted, uncontrollably inflammatory conditions that continue for months or even years, are caused by this intricacy (Landen et al., 2016; Jakovija and Chtanova, 2023). Furthermore, the local microenvironment around the wound may impact drug absorption, resulting in less than ideal therapeutic results (Kruse et al., 2015; Rodrigues et al., 2019; Raziyeva et al., 2021; Wang et al., 2022). As a result, novel materials and techniques is desperately needed to address and prevent several issues brought on by wound healing (Fang et al., 2023; Fang et al., 2024; Zhong et al., 2024).

Microneedle patches, which are made up of adhesive patches and hundreds of micron-sized needle arrays, present a novel mainstream method for transdermal medication delivery by gently penetrating the stratum corneum into the skin tissue (Jin et al., 2018; Chen et al., 2019a; Chen et al., 2020; Zhao et al., 2022). Microneedles are widely employed in the medical field for the treatment of skin diseases (Sabri et al., 2019), vaccine delivery (Prausnitz et al., 2009; Menon et al., 2021), insulin administration (Chen et al., 2018; Chen et al., 2019b), and stem cell transplantation (Chen et al., 2022) because of their special technological benefits. By avoiding problems with drug degradation by gastrointestinal enzymes and the first-pass action in the liver, microneedle delivery can reduce systemic side effects and improve drug absorption in comparison to conventional oral and injectable drug delivery methods. In contrast to conventional transdermal delivery techniques, microneedles offer a painless, non-invasive means of boosting medication absorption and increasing patient compliance (Bayda et al., 2019; Akhtar et al., 2020; Avcil and Celik, 2021; Jeong et al., 2021). Given these advantages, exploring the use of microneedles in wound healing becomes a compelling area of research.

Recent studies have demonstrated that microneedles have special benefits for accelerating wound healing. When it comes to wound dressings, microneedle tips are superior because they can more easily pass through physical barriers like scars, blood clots, and exudates at the wound site, hence preserving high local medication concentrations over time (Lyu et al., 2023). Additionally, by supporting the insertion site and causing collagen deposition and remodeling through mechanical stimulation (Aust et al., 2011; Busch et al., 2018), the array structure of microneedles can change the local stress environment, accelerating wound healing and minimizing scarring (Zhang et al., 2022). Microneedles are also commonly used in biosensing because when in direct contact with interstitial fluid at the wound site, their needles can detect glucose, uric acid, cholesterol, sodium ions, PH levels, and other markers with greater sensitivity and accuracy than other detection materials (Kolluru et al., 2019; He et al., 2021). Despite these promising results, there is still a need for further research to optimize microneedle applications for wound healing.

In addition to microneedles, enhancing the efficiency of drug delivery is crucial. As an additional tool to improve medication delivery rates and encourage wound healing, we employed an iontophoresis machine. Compared to passive diffusion, iontophoresis greatly increases the efficiency of medication delivery via the skin by facilitating the molecular transport of charged and neutral biomolecules across biological membranes through electrophoresis and electroosmosis. Iontophoresis has the ability to modify a number of cellular processes and functions, including as contraction, migration, and proliferation, by fostering interactions between different cellular constituents such ion channels, membrane-bound proteins, and the cytoskeleton (Alvarez et al., 1983; Jennings et al., 2008; Vieira et al., 2011). In the process of healing a wound, the endogenous electric field produced by iontophoresis can stimulate angiogenesis, augment local blood flow, and encourage fibroblast migration and collagen synthesis at the site of injury (Zhao et al., 2004; Clarke Moloney et al., 2006). These features make iontophoresis a valuable technique to explore in conjunction with microneedles for enhanced wound healing.

In this work, we introduce the combination of microneedle techniques and iontophoresis into a device named CELLADEEP in application for wound healing *in vivo*. CELLADEEP is a compact, patch-based anti-aging device from ROOTONIX that uses patented iontophoresis and drug delivery system technology for rapid, hands-free nutrient absorption through capillaries, minimizing skin damage from prolonged usage. The CELLADEEP device was first evaluated by using *ex vivo* human-derived skin tissue by Zhang et al. in the previous study (Zhang et al., 2024). In *ex vivo* model, the “CELLADEEP Patch” significantly improved skin moisture, elasticity, and collagen density while also reducing wrinkles and inflammation, as shown by enhanced COL1A1 and hyaluronan synthase three expression and decreased MMP-1 and IL-1β levels. In this study, we used Sprague-Dawley rats as models for skin injuries and utilized the previously described devices to assess their efficacy in aiding wound healing. By combining microneedle technology with iontophoresis, we aim to demonstrate the great potential of the “CELLADEEP Patch” in accelerating wound healing. Our research seeks to provide a comprehensive solution that addresses the current challenges in wound care and improves therapeutic outcomes.

## 2 Materials and methods

### 2.1 Materials

CELLADEEP and CELLADEEP Patches, containing Sodium Hyaluronate, Niacinamide, Retinol, Tuber Melanosporum Extract, Panthenol, Ethyl Ascorbyl Ether, Tocopheryl Acetate, Arbutin, Hydrolyzed Collagen, Melaleuca Alternifolia (Tea Tree) Leaf Extract, Acetyl Hexapeptide-8, and Adenosine, were supplied by ROOTONIX Company (Seoul, Republic of Korea). All chemicals used in this research were of high grade. There are approximately 2600 cone-shaped microneedles combined in the patch, and the patch is 9 cm*8 cm.

### 2.2 Animal experiment

Ten Sprague-Dawley rats weighing between 200 and 300 g, 8 weeks old, were utilized in the study. The animals were housed under controlled conditions with a 12-h light-dark cycle in a specific pathogen-free (SPF) environment, provided *ad libitum* access to food and water. The experimental procedures involving animals were conducted in accordance with the guidelines set forth by the Institutional Animal Care and Use Committee of Seoul National University Hospital (approval number: BA-2403-388-004) and adhered strictly to the NIH Guide for the Care and Use of Laboratory Animals.

The rats were randomly divided into two groups: the patch group (n = 5) and the untreated group (n = 5). Each group underwent a procedure where their dorsal hair was clipped after local anesthesia administration. The shaved area was disinfected with a povidone-iodine solution. Using a 10 mm diameter biopsy punch, a full-thickness circular wound was created on the dorsal skin of each rat. The untreated group did not receive any intervention, whereas the patch group received daily treatment using a therapeutic device for 5 minutes, followed by application of commercial film dressings (Tegaderm, 3M, Saint Paul, MN, USA) which were changed daily.

Photographs of the wounds were taken on days 3, 5, 7, 10, and 14 using a digital camera. After a 12-day treatment period, the rats were euthanized using CO2 hypoxia. Skin samples encompassing the entire thickness surrounding a 12 mm diameter circular wound were collected from each rat. These samples were fixed in 10% formaldehyde, dehydrated through a series of alcohol solutions (ranging from 80% to 100%), embedded in paraffin, and sectioned into 5 µm thick slices. The sections were subjected to various histological stains including Hematoxylin and Eosin (to assess granulation tissue and epidermal thickness), Masson’s trichrome (to evaluate collagen fiber production and density), and immunofluorescence staining.

### 2.3 Measurement of the wound healing area

The calculation for the closure of the wound area was determined using the following equation:

$$\text{Wound closing area }\left(\%\right)=\frac{A0\;‐\text{Ai}}{A0}\times 100\%$$

A0 and Ai represent the wound regions on day 0 and day i (0, 3, 5, 7, 10, and 14) respectively.

The A0 and Ai regions were acquired from microscope images using the ImageJ program.

### 2.4 Hematoxylin and eosin (H&E) staining

The tissue sections were deparaffinized in xylene for 3 min, followed by rehydration in decreasing concentrations of ethanol (100%, 95%, 90%, 80%, 70%) for 3 min each, and washed twice in distilled water for 3 min each. They were then stained with Hematoxylin for 5 min, rinsed in distilled water, incubated in Bluing reagent briefly, and stained with Eosin Y for 3 min. After dehydration in 100% ethanol baths for 3 min each, the sections were mounted with synthetic adhesive and covered. The H&E staining sections were observed and photographed with a digital microscope (AX10, ZEISS, Germany).

### 2.5 Masson’s trichrome (MT) staining

The tissue slides were stained following the manufacturer’s instructions: deparaffinization, rehydration, and Masson’s trichrome staining. Slides were incubated in Bouin solution overnight, rinsed with distilled water, stained with Weigert’s Hematoxylin for 10 min, washed, and immersed in Biebrich scarlet acid for 10 min. The sildes were then rinsed, stained in phosphomolybdic-phosphotungstic acid for 10 min, stained in aniline blue for 10 min, washed, and immersed in acetic acid for 3 min. The samples were dehydrated with 100% ethanol for 3 min, 95% ethanol for 2 min, and immersed in Xylene for 10 min. A drop of mounting media was added to each slide. Collagen fibers and intensity were observed with a microscope and quantified using ImageJ software (The National Institutes of Health, Bethesda, MD, USA).

### 2.6 Immunofluorescence staining

After deparaffinizing and hydrating the tissue slices in decreasing concentrations of ethanol (100%, 95%, 90%, 80%, 70%) for 3 min each, and washing twice in distilled water for 3 min each, 4% BSA in PBS was added and incubated at room temperature for an hour to prevent non-specific binding. The sections were then incubated overnight at 4°C with primary antibodies against vimentin, α-SMA, F4/80, MCP-1, VEGF, and CD68. The sections were washed with PBS, incubated for an hour at room temperature with Alexa Fluor 488 goat anti-mouse secondary antibody (goat anti-mouse IgG Alexa Flour 488®-conjugated secondary antibody (2090562, 1:1000, Invitrogen, Thermo Fisher Scientific, MA, USA), and then nuclear labeled with DAPI VECTASHIELD ((Fluoroshield with DAPI, Sigma-Aldrich, MO, USA) for fluorescence imaging. They were stored at −20°C and imaged using a confocal microscope (Zeiss LSM 710 (ZEISS group, Oberkochen, Germany). The intensity of indicators such as proliferation and inflammatory cytokines was measured using ImageJ software (The National Institutes of Health, Bethesda, MD, USA).

### 2.7 Protein preparation

On the fourteenth day, a RIPA lysis solution (Merck Millipore, Darmstadt, Germany) was prepared to extract proteins from the wound tissues of both untreated and patch groups. This solution containing Protease Inhibitor Cocktails (Roche Diagnostics, Laval QC, Canada) used to homogenize the tissues. The tissue extract was clarified by centrifugation at 14,000 rpm for 15 min at 4°C, and the supernatant was collected. The protein concentration was measured by using Pierce BCA Protein Assay Kit (Thermo Fisher Scientific Company, MA, USA) and stored at −80°C for future analysis such as Western blot, ELISA, ROS, and Nitrite assays.

### 2.8 Western blot

The expressions of α-SMA, collagen I, vimentin, TGF-β1, and β-actin in wound tissue during the proliferation phase were examined using a Western blot assay. The protein samples were run on SDS-PAGE gel and transferred on a nitrocellulose membrane. After blocking with 5% nonfat dry milk, the membranes were incubated overnight at 4°C with primary antibodies: mouse anti-α-SMA, mouse anti-vimentin, anti-GADPH, mouse anti-collagen I, β-actin (Santa Cruz Biotechnology, TX, USA), and mouse anti-TGF-β1 (Abcam, MA USA). The secondary antibody, an (H + L)-HRP conjugate (Biorad, CA, USA), was then applied for 2 hours. Protein expression levels were visualized using the ChemiDoc™ Imaging System (Bio-Rad Laboratories, CA, USA). Target protein expression was normalized to beta-actin expression using the ImageJ program.

### 2.9 ELISA

The inflammatory cytokine levels of TNF-α and IL-6 from day 14 purified protein were measured by ELISA Assay Kits (R&D System company, MN, USA).

### 2.10 Nitrite assay

The evaluation of nitrite and NO oxidation products in wound lysates was conducted using the Griess modified reagent. The protein was diluted by an equal amount using distilled water. A total of 100 µL of the 1× Griess modified reagent and 100 µL of the protein extract that had been diluted were left to incubate at room temperature for a duration of 15 minutes. Nitrite standard solutions of several concentrations were prepared to create a standard curve. The concentrations used were 1.6125, 3.125, 6.25, 12.5, 25, and 50 µM. The absorbance was measured at 405 nm using a BioTek Epoch 2 microplate spectrophotometer (BioTek Instruments, VT, USA).

### 2.11 ROS assay

ROS production was measured using DCF-DA kits (Abcam, United Kingdom). Protein extracts (30 mg/mL) from un-treatment and patch groups were mixed with 20 μM of DCF-DA solution and incubated at 37°C with 5% CO2 for 45 min in the dark. Fluorescence intensity was detected using Muti-microplate reader (SpectraMax id5, Avantor, PA, USA) with excitation/emission at 485/535 nm.

### 2.12 Statistics analysis

The statistical analysis was conducted by comparing the data among groups using GraphPad Prism 9 (GraphPad Software Inc, San Diego, CA, USA) software. The data were presented as the mean ± standard error of the mean (SEM) of five independent experiments. In the unpaired *t*-test, a *p*-value of less than 0.05 was considered statistically significant (*p < 0.05, **p < 0.01, ***p < 0.001).

## 3 Results

### 3.1 Macroscopic and microscopic visualization and observation of the wound healing process

**FIGURE 1:**
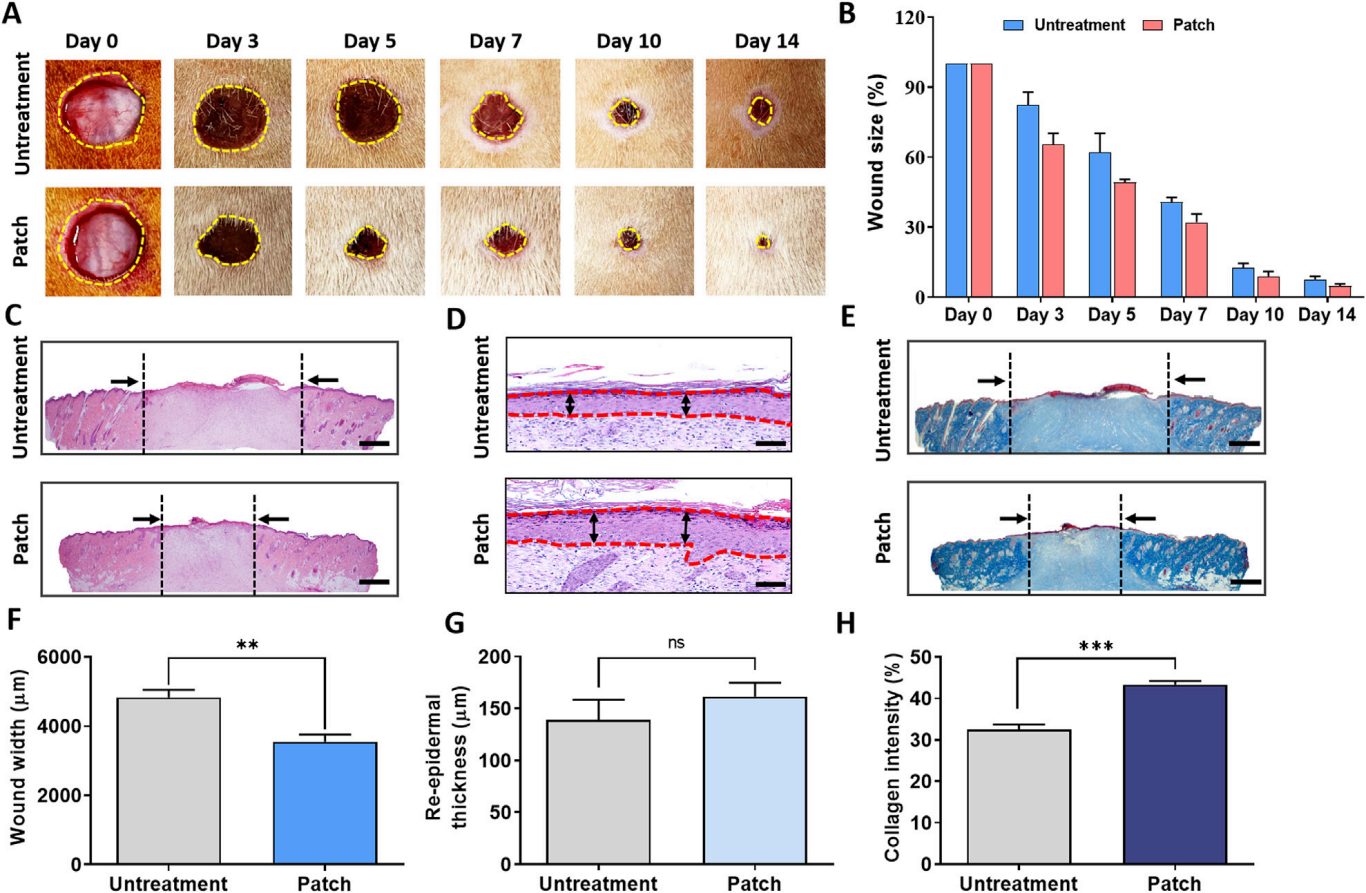
Enhancement of wound healing in rats treated with CELLADEEP patch. **(A)** Representative images of wound healing in the CELLADEEP patch-treated group compared to the untreated group. **(B)** Quantitative analysis of wound closure over time. **(C)** Hematoxylin and eosin (H&E) staining of cross-sections of epidermal layers on Day 14; scale bar: 1,000 μm. **(D)** H&E staining of cross-sections of epidermal layers on Day 14; scale bar: 50 μm. **(E)** Masson’s trichrome (MT) staining of wound sections on Day 14; scale bar: 1,000 μm. **(F)** Quantitative comparison of wound width on Day 14. **(G)** Quantitative comparison of re-epidermal thickness gap on Day 14. **(H)** Quantitative results of collagen density on Day 14. The data presented are empirical findings obtained from five rats in each group (n = 5). Results are shown as the mean ± SEM. Statistical significance is indicated as follows: **p < 0.01, ***p < 0.001.

The macroscopic images depict the progression of skin tissue on days 0, 3, 5, 7, 10, and 14 post-wound infliction. Throughout the wound healing process, both the untreated group and the patch-treated group showed a reduction in wound area. Notably, from the third day onwards, the patch-treated group exhibited a significantly smaller wound area compared to the untreated group (Figure 1A).

The bar chart on the right in Figure 1B illustrates the wound closure rate, expressed as a percentage of the initial wound area on day 0. It demonstrates that the patch-treated group experienced a notably faster rate of wound healing compared to the untreated group.

For microscopic evaluation of wound healing differences between the untreated and patch-treated groups on day 14, re-epidermalization and granulation tissue sections were stained with hematoxylin and eosin (H&E) and Masson’s Trichrome (MT), as shown in Figures 1C–E.

Histological analysis of H&E-stained cross-sections revealed a significant reduction in wound width in the patch-treated group (3,555 ± 200.5 μm) compared to the untreated group (4,831 ± 217.7 μm) (Figure 1F). Additionally, the epidermal thickness in the patch-treated group (161.5 ± 13.45 μm) was significantly greater than that in the untreated group (138.7 ± 19.63 μm) (Figure 1G).

MT staining further assessed collagen formation, showing markedly higher collagen deposition in the patch group compared to the untreated group, with the patch group displaying intense and uniform blue staining (Figure 1E). Quantitative analysis indicated that the blue density in the patch group was approximately 43.30% ± 0.9387%, significantly higher than the blue density of 32.56% ± 1.158% observed in the untreated group (Figure 1H).

### 3.2 Acceleration of the inflammatory phase and the inflammatory response during the wound healing process

**FIGURE 2:**
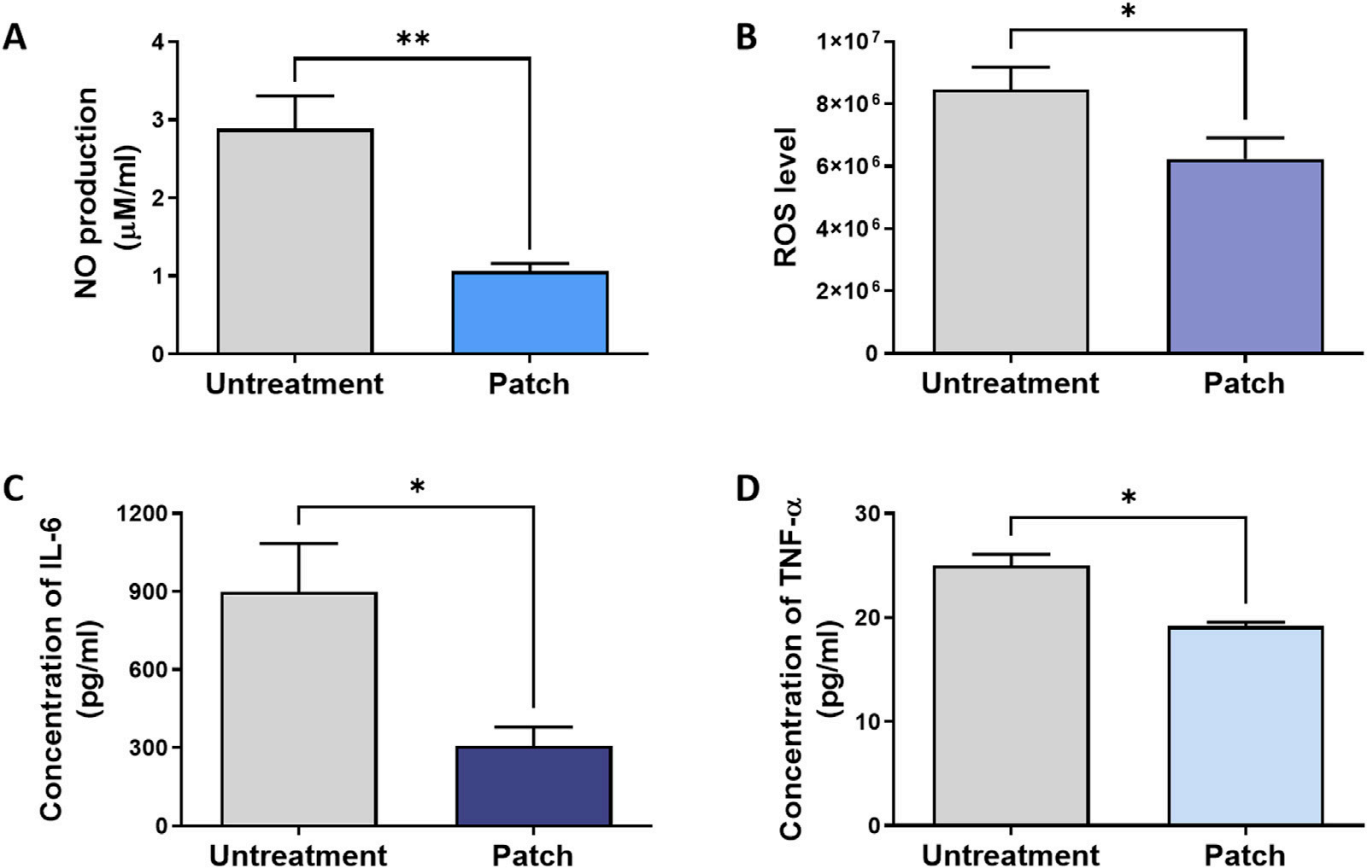
Attenuation of inflammatory response in rats treated with the CELLADEEP patch. Levels of pro-inflammatory biomarkers were analyzed in wound areas on Day 14: **(A)** Nitric oxide (NO) production, quantified using a nitrate assay. **(B)** Reactive oxygen species (ROS) levels, measured by ROS assay. **(C, D)** Concentrations of Interleukin 6 (IL-6) and tumor necrosis factor alpha (TNF-α), determined by ELSEA assays. The data presented are empirical findings obtained from five rats in each group (n = 5). Results are shown as the mean ± SEM. Statistical significance is indicated as follows: *p < 0.05, **p < 0.01.

As shown in Figure 2A, the nitric oxide (NO) level in the untreatment group reached 2.88 ± 0.42 μM/mL, whereas the NO level in the patch group was only 1.06 ± 0.10 μM/mL. The levels of reactive oxygen species (ROS) were also clearly distinguishable, with the patch group showing significantly lower ROS levels compared to the untreatment group (Figure 2B).

Additionally, ELISA assay was conducted at the conclusion of the healing process (day 14) to assess and quantify the expression of pro-inflammatory cytokines, evaluating the anti-inflammatory efficacy of the patch group (Figures 2C, D). The IL-6 level in the patch group (306.6 ± 72.82 pg/mL) was significantly lower than that in the untreatment group (898.0 ± 185.6 pg/mL) (Figure 2C). Similarly, the concentration of tumor necrosis factor-α (TNF-α) in the patch group (19.20 ± 0.3742 pg/mL) was lower compared to the untreated group (25.00 ± 1.095 pg/mL) (Figure 2D).

**FIGURE 3:**
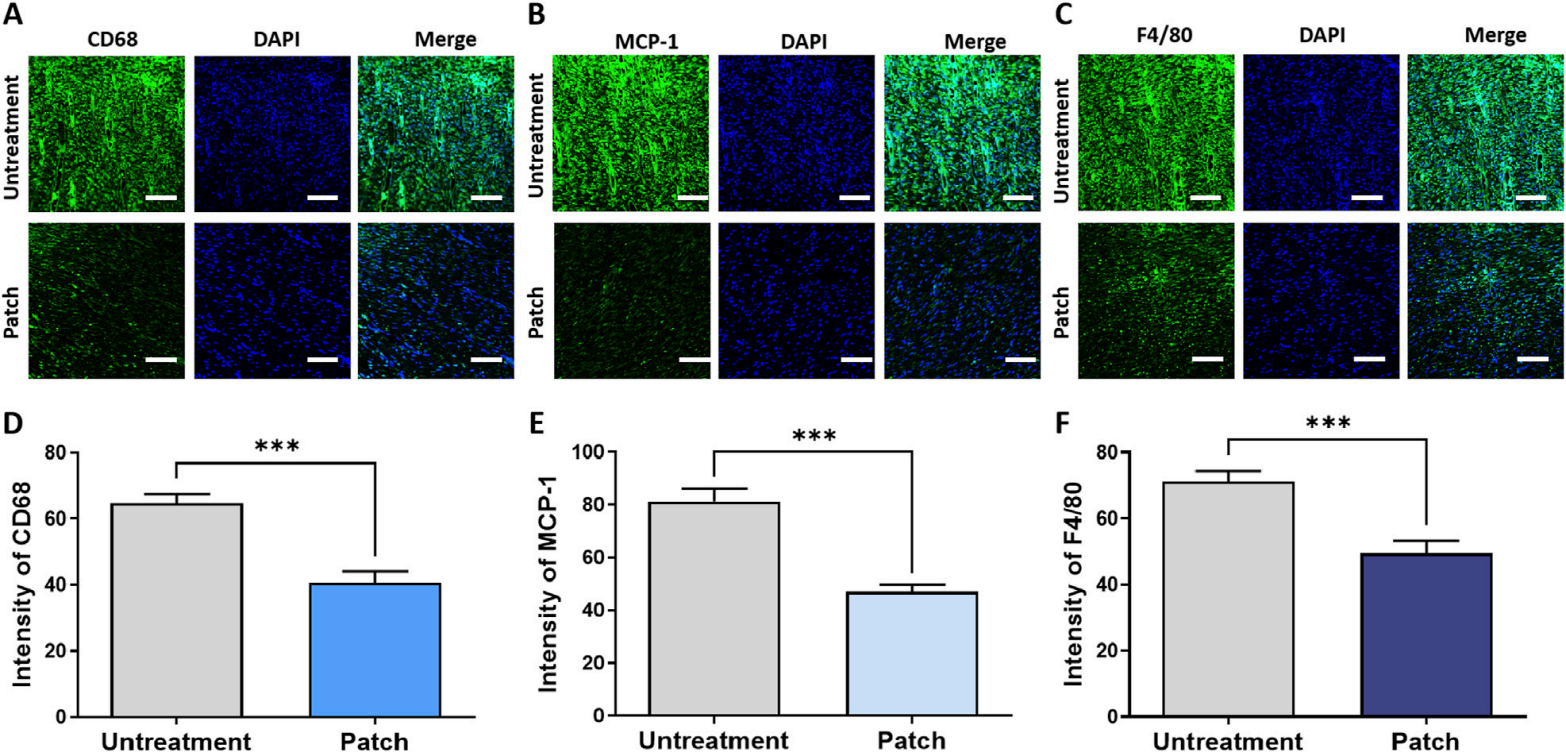
Attenuation of inflammatory response in rats treated with the CELLADEEP patch using immunofluorescence (IF) staining. **(A, D)** detection and quantification of CD68 in wounds without treatment and those treated with the CELLADEEP patch. **(B, E)** Immunofluoresce detection and quantification of Monocyte chemoattractant protein-1 (MCP-1) in wounds without treatment and those treated with the CELLADEEP patch. **(C, F)** Immunofluorescence detection and quantification of F4/80 in wounds without treatment and those treated with the CELLADEEP patch. The data presented are empirical findings obtained from five rats in each group (n = 5). Results are shown as the mean ± SEM. Statistical significance is indicated as follows: ***p < 0.001. Images were taken with scale bars: 50 μm.

To further validate these findings, immunofluorescence staining was performed to evaluate the inflammatory response and levels of pro-inflammatory cytokines in both the untreated and patch-treated groups. Quantitative analysis of the staining results demonstrated a significant decrease in the intensity of pro-inflammatory cytokines in the patch group compared to the untreated group, as depicted in Figures 3A–C. The expression intensity of CD68 in the patch group was 40.76 ± 3.203, while in the untreatment group it was 64.62 ± 2.826. The MCP-1 level was 47.06 ± 2.770 in the patch group, compared to 81.24 ± 4.988 in the untreatment group. The expression intensity of F4/80 followed a similar trend, increasing from 49.42 ± 3.852 in the patch group to 71.15 ± 3.311 in the untreatment group, with statistically significant differences (Figures 3D–F). These findings indicate that the CELLADEEP patch effectively enhances the wound healing process, accelerating its speed.

### 3.3 Acceleration of the proliferation phase and remodeling alteration during the wound healing process

**FIGURE 4:**
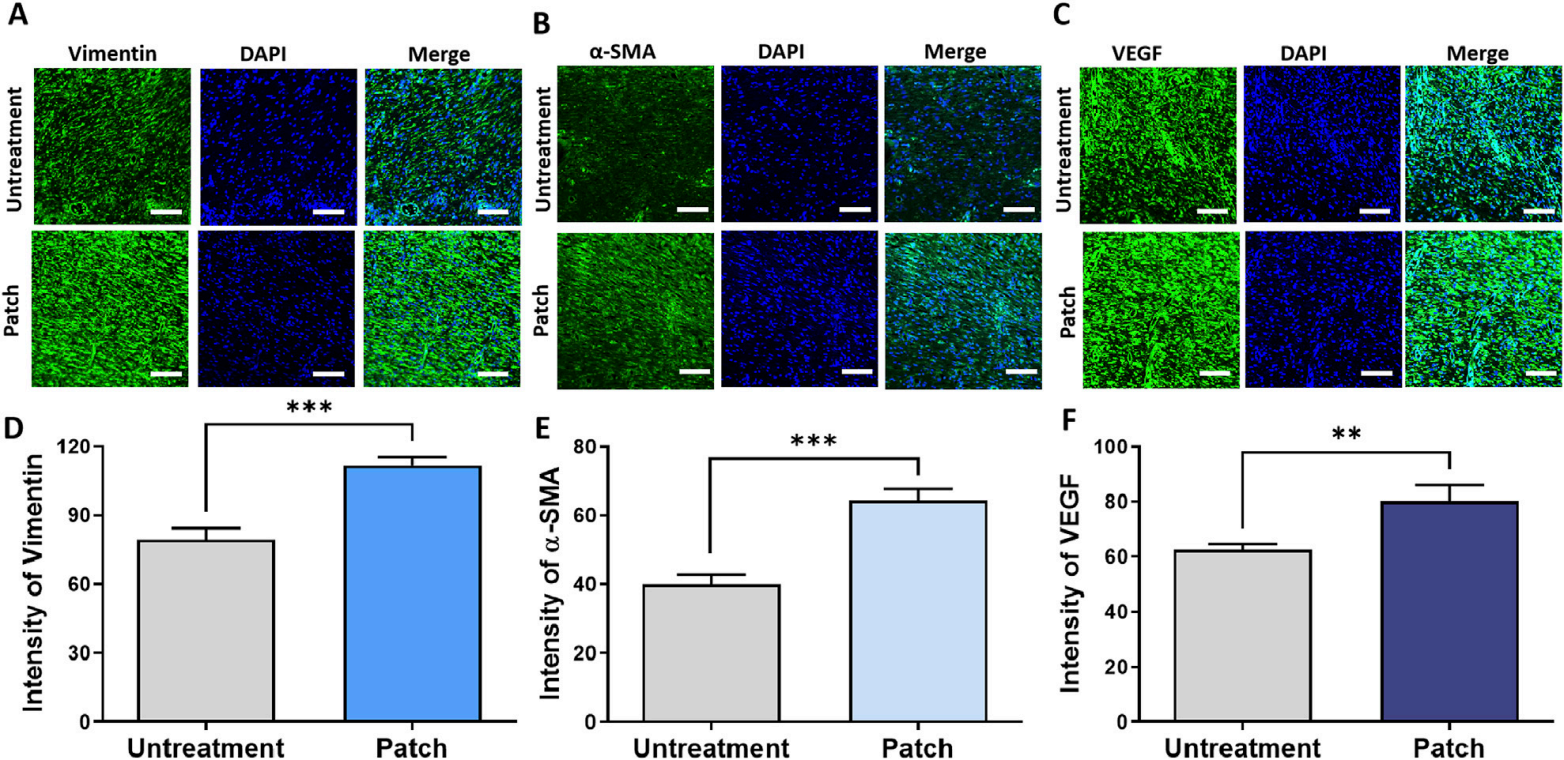
Enhanced proliferation in rats treated with the CELLADEEP patch by immunofluorescence (IF) staining. **(A, D)** Immunofluorescence detection and quantification of vimentin in wounds with and without treatment using the CELLADEEP patch. **(B, E)** Immunofluorescence detection and quantification of alpha smooth muscle actin (α-SMA) in wounds with and without treatment using the CELLADEEP patch. **(C, F)** Immunofluorescence detection and quantification of vascular endothelial growth factor (VEGF) in wounds with and without treatment using the CELLADEEP patch. The data presented are empirical findings obtained from five rats in each experimental group (n = 5). Mean values are shown with error bars representing the standard error of the mean (SEM). Statistical significance is indicated as follows: **p < 0.01, ***p < 0.001. Images were taken with scale bars: 50 μm.

To assess the proliferative effects of the CELLADEEP patch, immunofluorescence (IF) staining was conducted to detect various proliferation markers. The IF images in Figures 4A–C correspond to the markers such as Vimentin, α-SMA, and VEGF, respectively. Quantitative analysis of the staining results demonstrated that the fluorescence intensity of these proliferation markers was significantly higher in the patch group compared to the untreatment group. As shown in Figures 4D–F, the expression intensity of Vimentin in the patch group was 111.9 ± 3.394, while in the untreatment group it was 79.49 ± 4.917. The expression of α-SMA was 64.33 ± 3.384 in the patch group compared to 39.94 ± 2.869 in the untreatment group. Similarly, the expression intensity of F4/80 was 80.23 ± 5.808 in the patch group *versus* 62.57 ± 2.083 in the untreatment group, with these differences being statistically significant.

**FIGURE 5:**
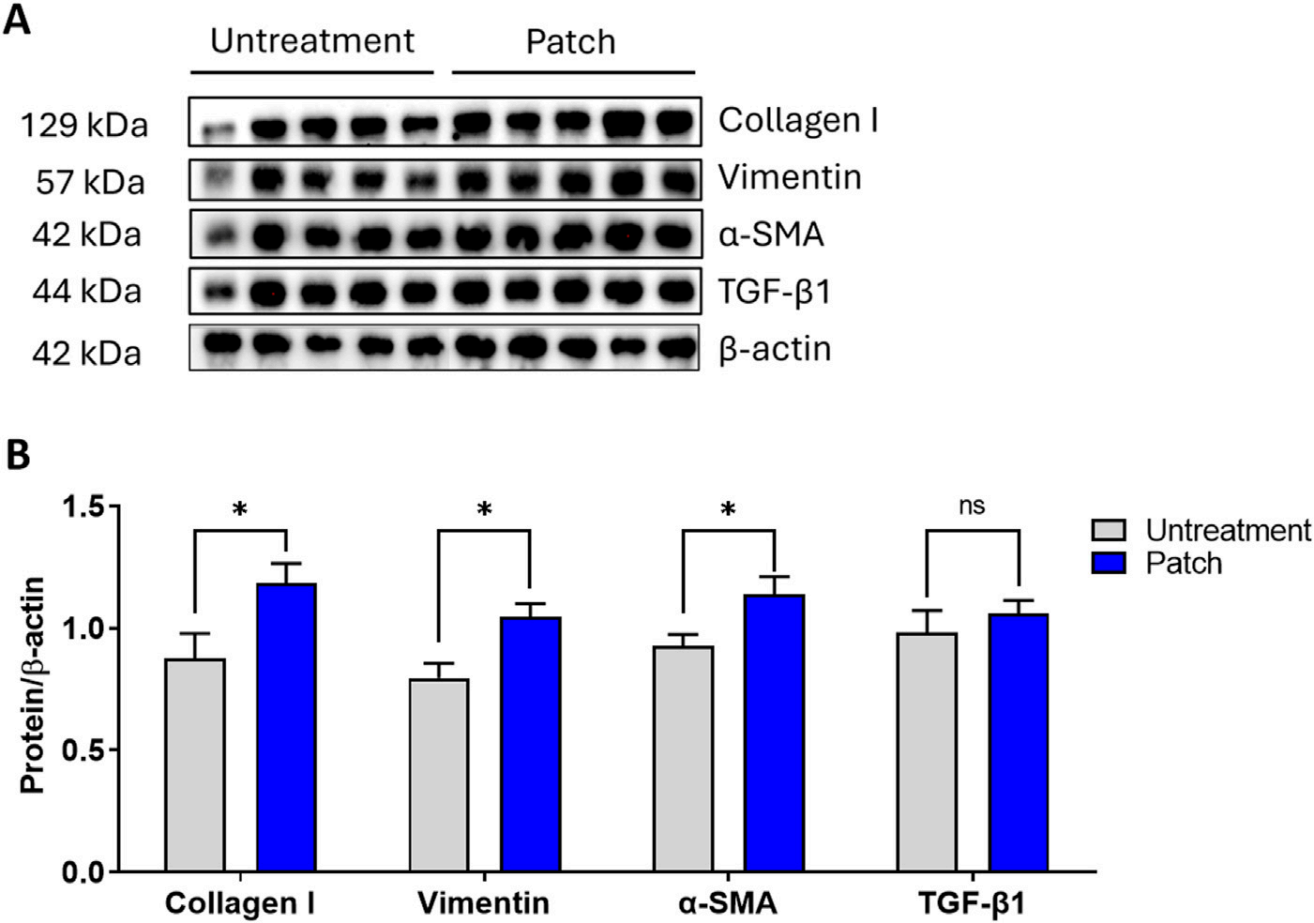
Enhanced proliferation in rats treated with the CELLADEEP patch revealed by Western blotting. **(A)** Western blot depicting changes in protein levels of collagen I, vimentin, α-SMA, TGF-β1, and β-actin between untreated and patch-treated groups on Day 14. **(B)** Densitometric values were normalized against their corresponding total protein levels and are presented as mean ± SEM from five rats in each group (n = 5).

Additionally, to provide a more comprehensive evaluation of the proliferative effects of the CELLADEEP patch, Western blot analysis was conducted. As shown in Figures 4G, H, the expression levels of Vimentin, α-SMA, collagen I, and TGF-β1 were higher in the patch group compared to the untreatment group (Figures 5A, B).

## 4 Discussion

Our study aimed to evaluate the efficacy of the CELLADEEP cosmetic device in promoting wound healing. This device synergistically integrates iontophoresis therapy and microneedle patches, which are infused with multiple active ingredients, offering significant advantages over conventional wound healing modalities. To comprehend the results obtained during the wound healing process, it is crucial to consider the four stages of wound healing: hemostasis, inflammation, proliferation (including granulation, angiogenesis, and wound closure), and remodeling. These stages are not discrete but rather occur in a dynamically overlapping manner (Guo and Dipietro, 2010). The healing process involves the spatiotemporal synchronization of various cell types, including inflammatory cells, fibroblasts, macrophages, keratinocytes, and endothelial cells, mediated by a milieu of released cytokines, chemokines, and growth factors (Barrientos et al., 2008; Tan et al., 2019).

During the wound healing process, combating inflammation involves recruiting immune cells to the wound site to counteract pathogens and clear debris, thereby releasing cytokines and growth factors that promote healing. IL-6 and TNF-α are key pro-inflammatory cytokines in this process. Elevated levels of TNF-α can lead to chronic inflammation and imbalance between pro- and anti-inflammatory responses (Schumacher and Naga Prasad, 2018), ultimately disrupting the natural healing process and causing delayed wound closure, impaired tissue regeneration, and reduced collagen production (Cheng et al., 2015; Mahmoud et al., 2024). Downregulation of their expression in our ELISA result (Figures 2C, D) indicates that CELLADEEP effectively alleviates excessive inflammatory responses. The observed decrease in cytokines associated with monocyte/macrophage activity, including CD68, F4/80, and monocyte chemoattractant protein-1 (MCP-1), as determined through Immunofluorescence staining, substantiates the anti-inflammatory effects. These markers are indicative of macrophage recruitment and activation, and their reduction suggests a transition towards a more regulated and less pro-inflammatory macrophage phenotype (Sim et al., 2022).

Additionally, CELLADEEP positively impacts nitric oxide (NO) production and reactive oxygen species (ROS) levels, which are critical for cellular signaling and defense mechanisms. NO is an endogenous gaseous mediator that plays a central role in the regulation of vascular homeostasis, inflammation, and antimicrobial action during the three main phases of wound healing. Overabundance of NO levels are linked to infected or highly inflamed wounds, leading to further tissue damage. The observed reduction in NO production in the nitrite assay indicates decreased inflammatory activity, beneficial for tissue remodeling (Malone-Povolny et al., 2019). The downregulation of ROS levels performed by ROS assay (Figure 2B) suggests that CELLADEEP helps maintain oxidative homeostasis, preventing the oxidative stress caused by exorbitant ROS levels, which can hinder normal tissue remodeling (Dunnill et al., 2017). By reducing ROS and NO levels in the tissue, CELLADEEP alleviates oxidative stress, maintains oxidative balance, and triggers cellular signaling and defense mechanisms. Overall, these findings suggest that CELLADEEP promotes a controlled inflammatory response and prevents chronic inflammation, which is essential for effective wound healing, as inflammation needs to resolve promptly.

During proliferation of wound healing, processes such as angiogenesis, collagen deposition, granulation tissue formation, and re-epithelialization occur. Macroscopic observation and HE staining revealed that the wound width decreased and epidermal thickness increased in the patch treatment group, indicating enhanced epithelial regeneration and granulation tissue formation (Figures 1A–C). Furthermore, MT staining showed a significant increase in collagen deposition in the patch treatment group, indicating improved extracellular matrix production and remodeling. While high collagen strength is necessary for restoring tissue integrity, an overproduction of strong collagen during wound healing can lead to fibrosis, where scar tissue becomes excessive, stiff, and non-functional (Wynn and Ramalingam, 2012; Singh et al., 2023). Effective wound healing depends on balancing collagen production, remodeling, and degradation to prevent fibrosis. Pro-inflammatory cytokines regulate immune cell functions and key stromal cells like keratinocytes and fibroblasts during skin regeneration, but their chronic elevation impairs healing in systemic disorders (Wu et al., 2017; Nosenko et al., 2019). Prolonged inflammation by IL-6 may cause fibrosis or excessive scarring (Johnson et al., 2020). However, in our study, IL-6 secretion was lower in the CELLADEEP-treated group than in the control group, as proved by ELISA assay. While the inflammatory cytokine expression was reduced in the treatment group in this study, we conclude that the increase in collagen intensity is related to the effectiveness of CELLDEEP in forming the ECM but not related to fibrosis.

Fibroblasts, the most abundant cell type responsible for producing extracellular matrix (ECM) proteins, play a critical role in scaffolding vessels, tissues, muscles, and other structures. They are integral to cell development and repair processes and are essential for wound healing. Vimentin and α-smooth muscle actin (α-SMA) are markers of fibroblast activity and differentiation, respectively. Increased levels of vimentin and α-SMA caused by the usage of CELLADEEP indicats enhanced fibroblast function, increased contraction ability, and elevated ECM protein synthesis activity (Darby et al., 2016; Coelho-Rato et al., 2024).

The increased levels of vascular endothelial growth factor (VEGF) also suggest improved angiogenesis, as VEGF is crucial for vascular permeability, angiogenesis, endothelial cell proliferation, and migration, providing essential nutrients and oxygen to the healing tissue (Barrientos et al., 2008; Losi et al., 2013). During the proliferative phase, granulation tissue is filled with fibroblasts, which secrete large amounts of extracellular matrix components, most importantly collagen-I (Hinz et al., 2012). Collagen-I contributes to wound elasticity and mechanical strength and serves as a matrix for cell proliferation, attachment, and differentiation, involved in functions such as cell migration, tissue repair, and regulation of resident and inflammatory cell functions (Gardeazabal and Izeta, 2024). Additionally, transforming growth factor-β (TGF-β) drives the chemotaxis of inflammatory cells and fibroblasts to the injury site and promotes angiogenesis, granulation tissue formation, and the deposition of extracellular matrix components such as collagen and fibronectin, providing better mechanical strength (Ramanathan et al., 2017). The increased expression of collagen-I and TGF-β1 proteins promotes new tissue formation and extracellular matrix remodeling. These results collectively suggest that CELLADEEP effectively promotes wound healing by enhancing fibroblast activity, angiogenesis, and extracellular matrix production, improving wound strength and integrity.

The combination of microneedle technology and iontophoresis in the CELLADEEP patch is also a key factor in its efficacy. Microneedles facilitate the delivery of active ingredients by creating microchannels in the skin, allowing for deeper penetration and sustained release of therapeutic agents, thereby increasing local drug concentrations (Lyu et al., 2023). The mechanical stimulation provided by microneedles can also promote collagen deposition and remodeling (Busch et al., 2018). Moreover, the CELLADEEP microneedle patch contains specific active ingredients such as sodium hyaluronate, collagen, niacinamide, and retinol, which contribute to these effects. Sodium hyaluronate is known for its moisturizing and tissue-repairing properties, promoting wound healing by enhancing mesenchymal and epithelial cell migration and differentiation, as well as angiogenesis and collagen deposition. Additionally, Sodium hyaluronate fosters a favorable wound healing environment and has significant potential in treating scar formation (Papakonstantinou et al., 2012; De Francesco et al., 2023). Collagen is a key component of most wound healing formulations under development, widely present in the extracellular matrix and connective tissues. Its physical properties, such as high tensile strength, adhesiveness, elasticity, and remodelability, play a vital role in the wound healing process (Sharma et al., 2022). Niacinamide and retinol enhance skin barrier function and promote cell renewal (Gehring, 2004; Mukherjee et al., 2006). The anti-inflammatory and antioxidant properties of tea tree leaf extract and ethyl ascorbyl ether further support the reduction of inflammatory markers and oxidative stress (Carson et al., 2006; Telang, 2013).

Iontophoresis is frequently used as an electrical device that applies a steady, low-voltage current and an electric field to drive charged molecules across the skin barrier, thereby enhancing transdermal drug delivery and improving its overall efficiency (Alvarez et al., 1983; Jennings et al., 2008; Vieira et al., 2011). Iontophoresis offers controlled, programmable delivery while also improving local blood flow, stimulating angiogenesis, and promoting collagen synthesis and fibroblast migration (Zhao et al., 2004; Clarke Moloney et al., 2006). Iontophoresis devices are appeared in the form of patches which are combined with a solution or gel like drug (Santos et al., 2018). The patches were applied in designs by Talbi et al., which allow for controlled drug delivery by varying current density; and the sol-gel patch system for varicose veins of Murari et al. utilizes iontophoresis to manage drug release and penetration through the skin (Murari et al., 2018; Talbi et al., 2018). Moreover, Shin et al. developed an all-hydrogel-based electronic skin (e-skin) patch, integrating various functional hydrogels with electric field stimulation and iontophoresis which accelerated wound healing *in vivo* (Shin et al., 2024). Another example of iontophoresis-combined application which aissits in wound healing was shown by the enhance of ethosomal piroxicam permeation by iontophoresis in *ex vivo* rat model by Kazemi et al. (Kazemi et al., 2019).

Iontophoresis technology has been combined with other enhancement techniques for better transdermal delivery effect. Microneedle systems utilize arrays of tiny needles to create microchannels in the skin through epidermis layer, allowing for the delivery of macromolecules and hydrophilic substances (Munch et al., 2017; Noh et al., 2018). When combined with iontophoresis, this approach significantly enhances the diffusion of hydrophilic and charged macromolecules by facilitating their transport through the low-resistance channels formed by the microneedles. For example, a microneedle wearable device with synergistic effect was designed by Zheng et al. which facilitated effectively control vaccine delivery by activating cell in epidermis and dermis layer by adjusting the iontophoresis current, demonstrating an efficient and controllable transdermal delivery both *in vivo* and *in vitro* (Zheng et al., 2023). Li et al. presented an iontophoresis-driven microneedle-arrays patch designed for patient-friendly, long-lasting transdermal delivery of liquid macromolecular drugs, which supports active drug administration and showed 99% skin penetration with minimal cytotoxicity and no irritation (Li et al., 2021). Therefore, based on these combined effects, CELLADEEP may help accelerate the wound healing process observed in this study.

Despite the encouraging results indicating the efficacy of the CELLADEEP patch in promoting wound healing in a rat skin injury model, several limitations and future challenges need to be addressed. For instance, long-term effects, including potential side effects or complications from prolonged use of the CELLADEEP patch, were not evaluated. Future studies should include long-term follow-up to assess these factors. Moreover, future research should prioritize the assessment of the patch’s effectiveness on various categories of wounds, such as chronic and diabetic ulcers.

## 5 Conclusion

Altogether, we evaluated the efficacy of the CELLADEEP patch in promoting wound healing. CELLADEEP significantly accelerates wound healing compared to untreated controls by incorporating microneedle technology and iontophoresis. By day 14, the CELLADEEP-treated group demonstrated faster closure, reduced wound area, enhanced epidermal regeneration, and improved extracellular matrix remodeling in treated wounds by histological assessment. Suppression of inflammation evidenced by decreased levels of NO, ROS, and pro-inflammatory cytokines indicates that CELLADEEP helps control excessive inflammation and oxidative stress. Additionally, the patch promoted cellular proliferation and tissue remodeling, as shown by elevated expression of Vimentin, α-SMA, and collagen. While the results are promising, further research is needed to evaluate the long-term effects and effectiveness of CELLADEEP in different types of wounds to fully assess CELLADEEP’s clinical applicability and safety.

## Data Availability

The raw data supporting the conclusions of this article will be made available by the authors, without undue reservation.
